# We cannot empathize with what we do not recognize: Perceptions of structural versus interpersonal racism in South Africa

**DOI:** 10.3389/fpsyg.2022.838675

**Published:** 2022-09-28

**Authors:** Melike M. Fourie, Samantha L. Moore-Berg

**Affiliations:** ^1^Centre for the Study of the Afterlife of Violence and the Reparative Quest, Stellenbosch University, Stellenbosch, South Africa; ^2^Annenberg School for Communication, University of Pennsylvania, Philadelphia, PA, United States

**Keywords:** empathy, empathic concern, perspective taking, interpersonal racism, structural racism, intergroup threat, competitive victimhood\keywordbelowspace-30pt

## Abstract

Recent research suggests holding a structural, rather than interpersonal, understanding of racism is associated with greater impetus to address racial disparities. We believe greater acknowledgment of structural racism also functions to mitigate against empathic failures in response to structural injustices. Given South Africa’s situatedness as a country characterized by historical racialized oppression and continuing unjust legacies, it is appropriate to examine these ideas there. Across three studies, we tested the hypotheses that members of advantaged groups’ perspective taking and empathic concern may be compromised in response to people challenging the unequal *status quo*, and that *a priori* perceptions about the impact of structural (vs interpersonal) racism may mitigate or exacerbate such empathic failures. In Study 1, a national sample of White South Africans (*n* = 195) endorsed perceptions of interpersonal racism more readily than perceptions of structural racism, and expressed high levels of competitive victimhood for perceived anti-White structural racism. Studies 2 (*n* = 138) and 3 (*n* = 85) showed that White participants at a historically White university responded with impaired perspective taking and intergroup empathy bias in response to people challenging structural disparities. Finally, reduced recognition of continuing structural racism predicted greater intergroup empathy bias, which, in turn, was associated with reduced willingness to engage in intergroup discussions about past harm (Study 3). We propose that greater acknowledgment of structural racism is necessary not only to surmount intergroup empathic failures, but also to transcend the socioeconomically unequal legacies of apartheid and beyond.

## Introduction

Countries around the world have been compelled to acknowledge pervasive systemic and racialized inequality as it was forced into view by the unequal fall-out of the COVID-19 pandemic and the resurgence of the Black Lives Matter movement in response to racialized police brutality. Yet, many do not respond to these inequities with recognition, empathy, and allyship. In fact, some members of advantaged groups respond to people challenging the *status quo* with condemnation or indifference – a pattern seen in South Africa (Smith, 2017; Claasen, 2020; McMichael, 2020).

While South Africa is known for its peaceful transition from apartheid to democracy (Asmal, 2000), it remains one of the most racially unequal societies worldwide (Chatterjee, 2019). During apartheid, the government imposed socially constructed, hierarchical racial labels on its citizens with the objective to elevate the White minority at the expense of their Black African, Coloured (people of diverse racial origins), and Indian counterparts (Posel, 2001; see also Fourie et al., 2022 for present-day effects). Some argue that in South Africa’s transitional negotiations for peace, unity and reconciliation among citizens took primacy over social justice for the oppressed (Bornman, 2006; Swartz, 2016), so that in time, hopes of broad structural change gave way to disillusionment amongst the economically disenfranchised (Hino et al., 2018). The 2015–2016 #FeesMustFall social justice movement is a powerful example, when Black^1^ students and allies mobilized nation-wide protests against the institutional forces that perpetuate apartheid-era inequalities (Albertus, 2019). While many White South Africans support social justice, in recent years there has been a surge in denialist right-wing ideologies (Van Zyl-Hermann, 2018). Why then, does empathy break down so readily during pivotal moments when the unequal *status quo* is being challenged?

Empathy is a key emotional process that can facilitate social change and healing in intergroup contexts (Stephan and Finlay, 1999; Fourie et al., 2013; Cehajic-Clancy et al., 2016). Empathic failures abound, however, driving not only unfavorable intergroup outcomes, such as discrimination and hostility, but also active conflict (Cikara, 2015; Fourie et al., 2017). Today we know that empathy is not automatic, but sensitively deployed based on *a priori* ideas and expectations as well as situational motivations (Zaki, 2014). Here we focus on empathic concern, which refers to other-oriented feelings of care and compassion when perceiving an individual in distress (Batson, 2009). In intergroup contexts, the difference in empathic concern felt for the ingroup versus the outgroup, referred to as “intergroup empathy bias” or “parochial empathy,” has been found to significantly predict negative intergroup interactions (Cikara et al., 2011; Bruneau et al., 2017).

Perspective taking, a cognitive route to understanding others, refers to the capacity to consciously adopt another’s point of view to imagine what they might be thinking and feeling (Batson et al., 1997; Stietz et al., 2019). It constitutes an effortful process whereby a person actively tries to imagine how another person is thinking and feeling−given the particular situation, how they respond to it, and what we know about their needs and desires (Barrett-Lennard, 1981; Batson and Ahmad, 2009). In this sense, perspective taking involves a sensitive, non-judgmental understanding of the other, whereby one is attuned to the way in which the other is affected by a situation.

Substantial evidence documents the efficacy of perspective taking in eliciting empathic concern, especially in intergroup contexts (Shih et al., 2009; Batson, 2011). Taking an outgroup member’s subjective perspective has the effect of endowing that individual with uniquely human mental qualities (Harris and Fiske, 2006). For example, narrative descriptions of outgroup individuals’ mental states attenuate intergroup empathy bias (Bruneau et al., 2015). By contrast, denying “mind” to outgroup members paves the way for moral disengagement and negative intergroup outcomes, including empathic failures (Čehajić et al., 2009; Kteily et al., 2015).

Of relevance for the present research is the relation between perspective taking and the propensity to recognize racial discrimination. Across a series of studies, adopting the perspective of an outgroup member engendered greater acknowledgment of the persistence of outgroup discrimination and support of actions to combat racial inequality (Todd et al., 2012). Likewise, predetermined political beliefs and ideologies have been shown to impact perspective taking and negative intergroup outcomes (Sparkman and Eidelman, 2016). Here we propose that the tendency of advantaged group members to acknowledge the impact of structural (vs interpersonal) racism is associated with the extent to which they take the perspective and feel empathic concern for those challenging situations of structural racism.

The dominant conceptualization of racism concerns interpersonal moral transgression, when individuals overtly discriminate or exercise prejudice against outgroup members during discrete behaviors (Ansell, 2004; Sommers and Norton, 2006; Unzueta and Lowery, 2008; Verwey and Quayle, 2012; Nelson et al., 2013). A more nuanced and complex conceptualization of racism involves covert institutional practices and structural factors (e.g., laws, policies, inherited wealth) that result in cultural, material, and symbolic advantages for some groups while marginalizing others (Ture and Hamilton, 1992; Lipsitz, 2011). Structural racism therefore concerns historical and ongoing systemic, race-based prejudice, which serves as one of the foundations for interpersonal racism, and perpetuates socioeconomic, health, criminal justice, educational, and ecological inequality (Salter et al., 2017).

Unfortunately, structural understandings of racism tend to be less commonly recognized by advantaged group members and/or believed to be something of the past because of various motivated psychological processes, e.g., the desire to view society as just and merit-based (Bonam et al., 2019; Kraus et al., 2019). In addition, structural factors may play a role in such misperceptions. For example, in South Africa White people may be less likely to hold accurate perceptions of the lived realities of Black (see text footnote 1) people because of less frequent intergroup contact across historically persistent patterns of residential segregation (Durrheim and Dixon, 2010).

Of significance, is that advantaged group members also stand to gain from a more willful denial of the impact of structural racism on disadvantaged groups (Steyn, 2012; Bonam et al., 2019). In fact, denial of discrimination and structural oppression is a worldwide phenomenon amongst advantaged group members despite abundant evidence attesting to its widespread effects (Pager and Shepherd, 2008). Advantaged group members might go as far as to claim that they are victims of structural inequality themselves, thereby maintaining their moral superiority and obstructing social change (Saguy et al., 2013; Young and Sullivan, 2016). This tendency to perceive one’s own group as having suffered more relative to an outgroup is known as competitive victimhood (Noor et al., 2008).

A theory that captures many of the above assumptions is that of system justification (Jost et al., 2004; Jost, 2019). Accordingly, people are motivated to view the social order as fair (to reduce internal conflict) and to maintain and justify existing social, economic, and political positions within society. Of significance to the present work is that system-justifying attitudes are frequently associated with distorted perceptions of the *status quo*, and situations threatening the system (e.g., protest action) may elicit defensive responses (Jost et al., 2013). Because a structural conception of racism raises awareness of White privilege and one’s implication in unjust systems, denial thereof serves the strategic goal of maintaining the racial *status quo* and with it access to tangible material advantages (Unzueta and Lowery, 2008; Milazzo, 2016). Indeed, recent research suggests holding a structural (vs. interpersonal) understanding of racism is associated with greater impetus to acknowledge and address existing racial disparities (O’Brien et al., 2009; Rucker et al., 2019; Rucker and Richeson, 2021). We believe that greater perceptions of structural racism also function to mitigate against empathic failures in response to structural injustices.

### Current research

Here we examined White South Africans’ perceptions about the prevalence of interpersonal versus structural racism, and whether such perceptions predicted empathic concern toward Black African individuals in multiracial scenarios portraying interpersonal or structural racism. We selected Black Africans as the targets of racism as they represent the largest group who suffered systematic discrimination during apartheid^2^ (Hino et al., 2018). While we acknowledge that the term racism should be reserved for race-based oppression by historically institutionalized systems, we also examined participants’ beliefs about racism directed against White people (henceforth “anti-White racism”; Saguy et al., 2013), given an increasing sense of perceived discrimination against White people (White “victimhood”) in South Africa (Van Zyl-Hermann, 2018).

We operationalized empathic concern as self-reported compassion for target individuals, and intergroup empathy bias as the difference in empathic concern reported for ingroup versus outgroup individuals. In a novel departure from previous research, we did not manipulate perspective taking as an experimental condition with instructions (e.g., imagine-other, imagine-self, or objective focus; see Stotland, 1969), but assessed whether participants took the perspectives of target individuals spontaneously (e.g., “What do you think about [target] behavior?”). We tested the following three main hypotheses.

First, we predicted that perceptions of racism by a White, national sample would be skewed, such that interpersonal racism would be acknowledged disproportionally more than structural racism (Study 1). The view that racism is lodged within individual minds tends to dominate lay beliefs about racism, is less threatening to group esteem, and might thus be easier to recognize (Sommers and Norton, 2006; Unzueta and Lowery, 2008). By contrast, structural understandings of racism tend to be less prevalent and its impact underestimated (Nelson et al., 2013; Milazzo, 2016; Bonam et al., 2019). Such misperceptions could be attributed to various motivated psychological processes and structural factors (Jost et al., 2013; Kraus et al., 2019). In addition, acknowledging the continuing impact of structural racism threatens White identity and privilege (Steyn and Foster, 2008). Therefore, we also predicted that competitive victimhood would be greater for structural compared to interpersonal racism.

Second, we reasoned that being able to take the perspective of another may not guarantee empathic concern but may be an important determinant of it. Hence, for reasons stated above, we predicted that White participants’ outgroup perspective taking in situations portraying Black individuals’ activism against structural racism will be compromised, and that their responses to such situations will be indicative of intergroup empathy bias (Studies 2 and 3). We did not predict such difficulties in perspective taking and empathic concern for interpersonal situations of racism and injustice. Finally, we hypothesized that perceptions of the prevalence of structural racism would predict empathic concern in situations where the unequal *status quo* is challenged, such that greater recognition of structural racism will be associated with reduced intergroup empathy bias (Study 3).

## Study 1

Here we assessed perceptions about the prevalence of interpersonal versus structural forms of racism in the lives of Black African and White South Africans. We predicted that White participants’ endorsement of interpersonal racism against Black African people would be greater than that of structural racism against Black African people.

### Methods

#### Participants

To determine the number of participants required to detect significant differences in perceptions of interpersonal and structural racism, we conducted an *a priori* power analysis for paired-samples *t*-tests using G*Power 3.1 (Faul et al., 2007). This analysis indicated that 199 participants would be required to detect a small within-group effect (*d* = 0.20) with 80% power. We recruited 200 South African participants identifying as White through a countrywide panel service to participate in an online survey assessing social attitudes (for additional details on sampling, see Supplementary material). We excluded five participants who did not complete all relevant questions, leaving a final sample of 195 (*M*_*age*_ = 39.62, SD_*age*_ = 12.12, 77% female, *M*_*yrs edu*_ = 13.67, SD_*yrs edu*_ = 3.07). Data were collected as part of a larger study that included additional measures not relevant to the present research question.

#### Measures

##### Demographics

Participants reported whether they are South African citizens, fluent in English, and identify as White (inclusion criteria), followed by their gender, age, and years of formal education.

##### Perceptions about racism

To assess perceptions of *interpersonal versus structural racism* against Black African and White South Africans, we first defined these terms to ensure that participants understood the distinction between them. *Interpersonal racism* was defined as “negative attitudes and discriminatory behaviors by individuals toward members of specific racial groups,” whereas *structural racism* was defined as “institutional practices and structural factors (e.g., laws, policies) that routinely disadvantage specific racial groups.” Participants were then asked the following six questions in counterbalanced order: “How much did [interpersonal/structural] racism affect Black African people during apartheid?” (anti-Black racism apartheid), “How much does [interpersonal/structural] racism affect Black African people today?” (anti-Black racism today), and “How much does [interpersonal/structural] racism affect White people today?” (anti-White racism today).^3^ These questions were adapted from Rucker et al. (2019), and were collected on continuous sliders ranging from 1 (*not at all*) to 100 (*very much*). The difference between participants’ perceptions of anti-Black and anti-White racism today was used as a proxy for competitive victimhood.

### Results

**FIGURE 1:**
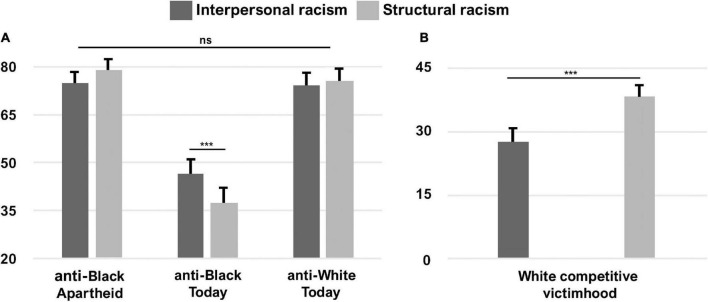
**(A)** Study 1 White participants’ perceptions of anti-Black interpersonal and structural racism during apartheid and today, and anti-White interpersonal and structural racism today. **(B)** Perceptions of competitive victimhood (anti-White racism today minus anti-Black racism today) were greater for structural than interpersonal racism. Error bars indicate standard error of the mean. ***p < 0.001. Panel **(A)** is reprinted by permission from Springer Customer Service Centre GmbH: Springer Nature, Affective Science (Fourie and Verwoerd, 2022). ns = not significant.

As hypothesized, White participants perceived anti-Black structural racism (*M* = 37.68, SD = 31.03) to have significantly less of an impact than anti-Black interpersonal racism (*M* = 46.74, SD = 30.52) on Black African people today, *t*(193) = 5.86, *p* < 0.001, *d* = 0.42. This is in contrast to their perceptions of anti-Black racism during apartheid, which was not only much greater than today, but anti-Black structural racism (*M* = 79.20, SD = 21.92) was also perceived to be more significant than anti-Black interpersonal racism (*M* = 74.97, SD = 24.62) during that time, *t*(194) = –3.57, *p* < 0.001, *d* = 0.26. By comparison, White participants perceived no significant difference between anti-White structural (*M* = 75.46, SD = 27.87) and anti-White interpersonal (*M* = 74.27, SD = 27.04) racism today, *t*(194) = –0.89, *p* = 0.376, *d* = –0.06 (Figure 1A).

When comparing perceptions of anti-Black and anti-White racism, it is noteworthy to observe that perceptions of anti-White interpersonal and structural racism today were significantly greater than that experienced by Black African people today (Interpersonal racism: *t*(193) = 8.93, *p* < 0.001, *d* = 0.64; Structural racism: *t*(193) = 11.98, *p* < 0.001, *d* = 0.86). Moreover, perceptions of anti-White interpersonal and structural racism today did not differ significantly from anti-Black interpersonal and structural racism experienced by Black African people under apartheid (Interpersonal racism: *t*(194) = –0.31, *p* = 0.757, *d* = –0.02; Structural racism: *t*(194) = –1.64, *p* = 0.102, *d* = –0.12). Such heightened perceptions of racism experienced by the historically advantaged ingroup (i.e., anti-White racism today minus anti-Black racism today) could be described as perceptions of competitive victimhood. In line with our hypothesis, in the present sample, competitive victimhood for perceived structural racism was significantly greater than competitive victimhood for perceived interpersonal racism, *t*(193) = –5.15, *p* < 0.001, *d* = –0.37 (Figure 1B).

### Discussion

As we anticipated, White South Africans in our sample perceived the impact of structural racism to be significantly less severe than interpersonal racism on Black African people’s lives today, despite structural racism’s stronger connection to deepening socioeconomic inequality and low social mobility than interpersonal racism (Day and Fiske, 2017; Salter et al., 2017).

White participants reported significantly inflated perceptions of anti-White racism today—on a par with that experienced by Black African people during apartheid—even though the continuing staying power of White privilege post-apartheid is recognized widely (Steyn and Foster, 2008; Chatterjee, 2019). These results could be explained through the lens of competitive victimhood, with White people not only underestimating the pervasive and continuing impact of structural forms of oppression that Black people experience, but also perceiving themselves to be at the brunt of racial oppression (Noor et al., 2017; Bentrovato et al., 2020).

Power disparities can shape how competitive victimhood processes play out (McNeill and Vollhardt, 2020). Advantaged group members may engage in competitive victimhood as a coping mechanism to deal with accusations of inflicting unjust suffering on disadvantaged groups and to restore their group’s moral integrity (Sullivan et al., 2012). While feelings of competitive victimhood may not be a conscious process, it assuages guilt through retrospective justification. Hence, by claiming relative victim status, people of advantaged groups can reduce feelings of responsibility for previous harmdoing and defend their positive identity (Tov-Nachlieli et al., 2013; Young and Sullivan, 2016). Competitive victimhood also strengthens identification with the ingroup, and has been associated with reduced outgroup empathy (Noor et al., 2008).

Taken together, as in the US (Kraus et al., 2017, 2019), White South Africans in our sample considered the impact of more pervasive (albeit less explicit) structural forms of racism on Black African people to be significantly less than that of interpersonal forms of anti-Black racism today. Such rationalizations are costly as they may serve to justify and stabilize unequal systems (Jost et al., 2013). Notably, distorted perceptions about structural racism, combined with feelings of competitive victimhood, may limit White people’s motivation to recognize and respond with empathic concern to Black people challenging structural oppression. Study 2 tests this hypothesis.

## Study 2

Here we examined perspective taking and empathic concern in response to ecological scenarios about structural and interpersonal racism involving Black African and White individuals. We predicted that White participants (i) would not readily take the perspectives of Black individuals who challenge structural racism, and therefore respond with significant intergroup empathy bias, but (ii) would take the perspectives of Black individuals in interpersonal situations of racism or injustice and respond with high empathic concern. We hypothesized that Black African participants would respond with high perspective taking and empathic concern for Black individuals in all situations of racism or injustice.

### Methods

#### Participants

To determine the number of participants required to detect significant differences in ingroup and outgroup empathic concern, we conducted an *a priori* power analysis for paired- samples *t*-tests using G*Power 3.1 (Faul et al., 2007). This analysis indicated that 90 participants would be required to detect small-medium within-group effects (*d* = 0.30) with 80% power. We recruited 247 undergraduate students from lecture halls at a historically White public university to complete study procedures. Because we were interested in White and Black African participant responses, we excluded 80 individuals who identified as either foreign nationals (*n* = 13), Coloured, Indian or Asian (*n* = 64), or who did not provide demographic information (*n* = 3). The final sample consisted of 167 participants (*M*_*age*_ = 19.39, SD_*age*_ = 1.37, 82% female, *M*_*yrs edu*_ = 13.11, SD_*yrs edu*_ = 0.98, 138 White, 29 Black African). Although the Black African sample was comparatively small, based on our obtained within-group effect sizes (scenario 1: *d* = 0.49, scenario 2: *d* = 1.56, scenario 3: *d* = 0.96), we achieved β > 0.83. We therefore include their responses to the scenarios here as comparison to those of White participants.

#### Measures

##### Reflective scenarios

Participants were asked to read and respond to three short multi-racial scenarios including White and Black individuals. To ensure that the scenarios appeared authentic with high ecological validity, they were modeled after real events. Each scenario was furthermore framed to allow perspective taking for both parties involved, thus avoiding an apparent “wrongdoer” and “victim” which would dictate responses. Scenario 1 involved a protest scene at the university that participants attended, where Black workers expressed their frustration toward a statue which symbolizes Black oppression and perpetuation of the outsourcing system, while White students silenced the Black workers’ response. Scenario 2 involved a situation of interpersonal injustice between a Black domestic worker and her White employers. Scenario 3 involved a situation of interpersonal racism common in sports. The three scenarios were similar in length and presented in the following order:

*Scenario 1: (opposition to) Structural racism.* Last year an incident was reported to the University Transformation Office: During a protest by the university’s outsourced workers, a few workers threw mud on the J.H. Marais statue (a heritage treasure). Some White students then decided to do something about the situation. They went to the statue with cleaning materials and started to clean up the mess. The cleaning of the statue by the students angered the workers, however. Soon a cycle started where workers threw more and more mud on the statue and the students kept cleaning it without backing off.

*Scenario 2: Interpersonal injustice.* Nomusa is a domestic worker who has worked at the Eksteen household for the past two years. Recently Nomusa suffered a great loss: her house burned down as a result of an accident involving tile glue and she lost most of her possessions. Two months after the incident she asked her well-to-do employers, Mr. and Mrs. Eksteen whether they would help with an expensive dress for her daughter’s matric farewell. The dress cost ZAR800 *(approximately US $50)*. While Mr. and Mrs. Eksteen have been supportive following the accident, they refused to help Nomusa with money for the dress.

*Scenario 3: Interpersonal racism*. The following scenario was reported at an elite secondary girls’ school in Cape Town. Zoliswa is a talented athlete and recently went to the national hockey championships where she was selected for the National Hockey Team, a very prestigious achievement. Her initial excitement was dampened, however, when some of the White players on the team acted unfriendly/hostile toward her. When she came home, she told her parents, to their utter dismay and disbelief, that she would not take up the position as she did not want to be seen as a “quota player.”

*Spontaneous perspective taking* was assessed following each scenario through two free-text questions that did not prime participants to imagine each target’s motivation. Participants were asked to indicate in their own words “What do you think about [White target behavior]?” in the scenario and “What do you think about [Black target behavior]?” in the scenario. Responses to each target in each scenario were deductively coded as perspective taking present (perspective taking = 1) or absent (perspective taking = 0). Specifically, a response that suggested “responsively knowing” or a “sensitive understanding” of a party’s internal state was scored positive for perspective taking (Batson and Ahmad, 2009). Imagine-self and imagine-other perspectives were both coded as perspective taking present (Todd et al., 2011). Two independent researchers blind to participants’ identity coded these data. Responses that were unclear/ambiguous in terms of perspective taking were omitted from data analysis (coded as missing data). The intercoder reliability values for scenarios 1 and 2 were moderate to strong (Kappa = 0.75 and Kappa = 0.82, respectively, *p*s < 0.001), whereas the intercoder reliability value for scenario 3 was somewhat weaker (Kappa = 0.69, *p* < 0.001; McHugh, 2012; Blick et al., 2018). Instances of intercoder disagreement were resolved through a consensus approach where discrepancies were discussed with the PI and mutual decisions reached (O’Connor and Joffe, 2020).

##### Self-reported emotion

In response to the Black and White targets in each scenario, participants were asked to indicate “How did [White/Black target behavior] make you feel?” Participants then rated the following emotions on a 1 (*not at all*) to 9 (*very much*) Likert-type scale: frustration, compassion, anger, pride, shame, and anxiety. Empathic concern was operationalized as compassion to ensure that participants understood its meaning. We created a composite *negative emotion* score that consisted of frustration and anger ratings (αs > 0.77).

### Results

#### Perspective taking

White participants’ perspective taking in response to Black target individuals was much less common in response to scenario 1 (structural racism: 27%) than scenario 2 (interpersonal injustice: 84%) or scenario 3 (interpersonal racism: 55%; Table 1). However, most White participants took the perspective of White target individuals in scenario 1 (93%), and less so in scenarios 2 and 3 (57% and 7%, respectively). By comparison, Black African participants readily took the perspectives of Black target individuals in scenarios 1 and 2 (82% and 88%), and to a lesser extent in scenario 3 (54%; Table 1). Examples of perspective taking (present/absent) qualitative responses can be found in Supplementary Table 1.

**TABLE 1:** Perspective taking frequency scores: Study 2.

	White participants (*n* = 138)		Black African participants (*n* = 29)	
		
	White target	Black target	White target	Black target
**Scenario 1: Structural racism** PT = 0 PT = 1 Missing data	9 (7%) 113 (93%) 16	92 (73%) 34 (27%) 12	12 (46%) 14 (54%) 3	5 (18%) 23 (82%) 1
**Scenario 2: Interpersonal injustice** PT = 0 PT = 1 Missing data	54 (43%) 73 (57%) 11	20 (16%) 105 (84%) 13	16 (60%) 11 (40%) 2	3 (12%) 23 (88%) 3
**Scenario 3: Interpersonal racism** PT = 0 PT = 1 Missing data	114 (93%) 8 (7%) 16	53 (45%) 66 (55%) 19	28 (100%) 0 (0) 1	13 (46%) 15 (54%) 1

Data presented are frequencies with percentage values in brackets. Missing data include unanswered questions and responses where the presence/absence of perspective taking was unclear. PT = perspective taking; 0 = absent; 1 = present.

White and Black African participants’ somewhat reduced perspective taking for the Black target in scenario 3, a more obvious situation of racism, was unexpected. Closer inspection of participant responses suggested that perspective taking in some instances were masked by participants’ reactive expression of negative emotion (despair) in response to Zoliswa’s decision to quit the team and “give in” to the White players. In their responses they therefore neglected to responsively consider *her* feelings and motivations. Indeed, self-reported negative emotion (frustration and anger) did not differ significantly from empathic concern for Zoliswa for either Black African (*p* = 0.545, *d* = –0.13) or White (*p* = 0.224, *d* = 0.11) participants (see Supplementary Table 2).

#### Self-reported empathic concern

Self-reported emotion ratings and statistical analyses to determine which emotions were felt most strongly in response to each target can be found in the Supplementary material (Supplementary Table 2). Below we focus on participants’ empathic concern responses.

**FIGURE 2:**
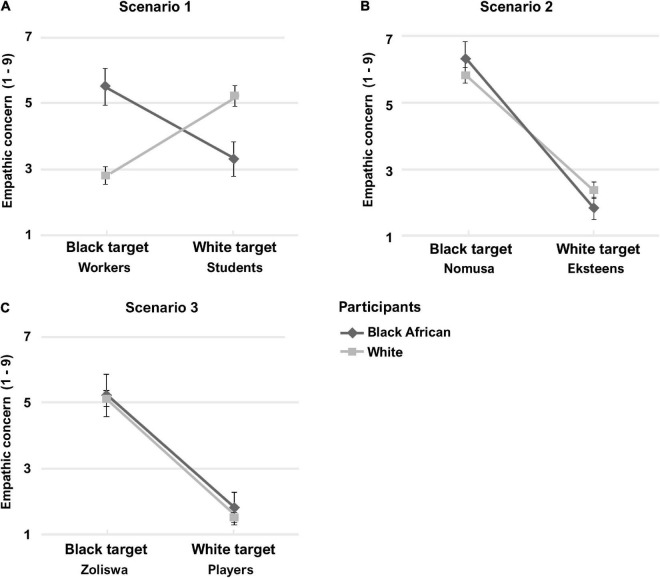
Study 2 self-reported empathic concern ratings for **(A)** scenario 1 (structural racism), **(B)** scenario 2 (interpersonal injustice), and **(C)** scenario 3 (interpersonal racism). Error bars indicate standard error of the mean.

For scenario 1 (structural racism), a paired-samples *t*-test indicated that White participants displayed significant intergroup empathy bias: empathic concern ratings were higher in response to White (*M* = 5.17, SD = 2.26) than Black (*M* = 2.90, SD = 2.00) target individuals, *t*(124) = –7.80, *p* < 0.001, *d* = 0.70. As anticipated, Black African participants also showed significant intergroup empathy bias, with empathic concern rated higher in response to Black (*M* = 5.17, SD = 2.62) than White (*M* = 3.30, SD = 2.49) target individuals, *t*(22) = 2.36, *p* = 0.028, *d* = 0.49 (Figure 2A).

For scenario 2 (interpersonal injustice) and 3 (interpersonal racism), White participants’ responses were reversed: they were more empathetic toward the Black (scenario 2: *M* = 5.85, SD = 2.32; scenario 3: *M* = 5.19, SD = 2.56) than White (scenario 2: *M* = 2.40, SD = 1.96; scenario 3: *M* = 1.59, SD = 1.36) target individuals, *t*(123) = 12.59, *p* < 0.001, *d* = 1.13 and *t*(115) = 14.15, *p* < 0.001, *d* = 1.31, respectively. Likewise, Black African participants were more empathetic toward the Black (scenario 2: *M* = 6.28, SD = 2.37; scenario 3: *M* = 5.35, SD = 2.96) than White (scenario 2: *M* = 1.83, SD = 1.43; scenario 3: *M* = 2.13, SD = 2.60) target individuals in these scenarios, *t*(22) = 7.48, *p* < 0.001, *d* = 1.56 and *t*(21) = 4.50, *p* < 0.001, *d* = 0.96, respectively. In fact, White and Black African participants’ empathic concern ratings did not differ significantly from each other for either White or Black target individuals in scenarios 2 and 3 (Mann–Whitney *U* tests < 1.40, *p*s > 0.16; Figures 2B,C).

#### Perspective taking and empathic concern

To determine how well perspective taking predicted empathic concern, we performed a simultaneous regression for each target in each scenario, with perspective taking (absent/present) scores and participant race as predictor variables, and target empathic concern ratings as the dependent variable. We inspected regression coefficients using 95% confidence intervals (CIs) derived through bootstrapping running 1,000 iterations (Efron, 1987; see Table 2).

**TABLE 2:** Simultaneous regressions predicting empathic concern as a function of perspective taking and participant race: Study 2.

	Scenario 1 Empathic concern		Scenario 2 Empathic concern		Scenario 3 Empathic concern	
			
	White target *R*^2^ = 0.14, *p* < 0.001	Black target *R*^2^ = 0.36, *p* < 0.001	White target *R*^2^ = 0.09, *p* = 0.002	Black target *R*^2^ = 0.20, *p* < 0.001	White target *R*^2^ = 0.16, *p* < 0.001	Black target *R*^2^ = 0.06, *p* = 0.024
Perspective taking	1.57** [0.20, 2.73]	2.34*** [1.55, 3.12]	1.04*** [0.47, 1.60]	2.74*** [1.55, 3.87]	2.54*** [0.96, 4.08]	1.24** [0.33, 2.15]
Participant race	1.54** [0.13, 2.82]	–1.36** [–2.55, –0.11]	0.55 [–0.04, 1.10]	–0.38 [–1.31, 0.68]	–0.81 [–2.02, 0.06]	–0.15 [–1.33, 1.17]

Data presented are unstandardized beta (B) values, with 95% bootstrap confidence intervals (CIs) in brackets. Participant race was coded as 1 = Black African, 2 = White. **p < 0.01. ***p < 0.001.

These analyses indicated that perspective taking significantly predicted empathic concern for both targets in scenario 1 (Black target: β = 0.48, *p* < 0.001; White target: β = 0.22, *p* = 0.010), scenario 2 (Black target: β = 0.44, *p* < 0.001; White target: β = 0.27, *p* = 0.001), and scenario 3 (Black target: β = 0.23, *p* = 0.006; White target: β = 0.37, *p* < 0.001). Participants who took a target’s perspective were thus much more likely to also respond with greater ratings of empathic concern. Of significance, is that participant race predicted empathic concern only for targets in scenario 1 (Black target: β = –0.22, *p* = 0.005, White target: β = 0.23, *p* = 0.008), but not those in scenarios 2 or 3. These findings corroborate our hypothesis, namely that White and Black African participants’ empathic concern responses would differ particularly for the scenario characterized by structural racism.

It should be noted that due to the cross-sectional, correlational nature of our study we could not determine causal relations between perspective taking and empathic concern, and that it is also plausible that empathic concern led to greater perspective taking (see Supplementary Table 3).

### Discussion

Consistent with our theorizing, taking an outgroup member’s perspective significantly predicted empathic concern for that individual. White participants were unlikely to take the perspective of Black individuals challenging structural racism (scenario 1) and responded with significant intergroup empathy bias, but readily took the perspectives of Black individuals in interpersonal situations of injustice (scenarios 2 and 3) and responded with high empathic concern. By contrast, Black African participants responded with high perspective taking and empathic concern toward Black individuals in each scenario. For scenario 1, in particular, their qualitative responses supported the interpretation that they understood that “*the workers’ protest action was directed at University management, not ‘White Students’, who saw it fit to defend a demeaning system*” (Black African participant; for more qualitative responses, see Supplementary Results).

Of significance, is that White participants did not fail systematically in all outgroup perspective taking and empathic concern responses. Rather, the context markedly impacted their responses, with a greater likelihood to express empathy for Black individuals when they were in contexts representing interpersonal (scenarios 2 and 3) than structural (scenario 1) racism. Understanding the mechanism(s) that underlie such failures in perspective taking and empathic concern is necessary to address and/or prevent them. Here we theorized that the type of racism (structural vs. interpersonal) plays a key role in motivating outgroup empathy. Below we consider support for this interpretation from the intergroup literature.

First, because structural racism is less about individual behavior and more about collective practices that produce unequal outcomes for different racialized identities, it is more likely to involve groups than interpersonal racism. Intergroup contexts (such as scenario 1), however, may provoke significantly more competition and aggression than interpersonal contexts (such as scenarios 2 and 3), thereby diminishing the motivation to care about an outgroup member’s misfortune (Meier and Hinsz, 2004; Cikara et al., 2011). Explicit competition can also increase the salience of social identity and connection with the ingroup (Hewstone et al., 2002), with potentially damaging effects for socially distant others (Tarrant et al., 2012; Waytz and Epley, 2012). Hence, because situations of structural racism are more likely to involve groups, they may be associated with increased intergroup empathy bias.

Second, structural racism might be threatening to White people’s self-image of being morally “good.” Unlike interpersonal racism, where the “bad” culprit is identifiable, structural racism increases awareness of White privilege and imparts a broader moral implication to the problem of racism (Unzueta and Lowery, 2008; Bonam et al., 2019). Scenario 1 may thus have posed a social identity threat to White participants, which, when disidentification with the ingroup is not possible, may be associated with defensive reactions (Branscombe et al., 1999; Halabi et al., 2008). The threat posed by scenario 1 could also be material, however, because participants’ *own* institution was at stake. Their empathy failures, therefore, might have been motivated by the desire to avoid loss of valuable resources (Riek et al., 2006; Zaki, 2014), whereas scenarios 2 and 3 did not involve personal costs. To rule out this possibility, we changed the context of scenario 1 to a different institution in Study 3.

Finally, opposition to structural racism is usually set in the public discourse around race and therefore highlights ideological discrepancies between groups, termed symbolic threat (Stephan et al., 2009). The relationship between perceived threat to the dominant (White) cultural worldview and increased racially biased outcomes is well documented (Greenberg and Kosloff, 2008; Craig and Richeson, 2014). In scenario 1, the ideological discrepancy involves beliefs of structural inequity, which was the reason for the workers’ protest, and which the White students silenced with their behavior. White participants might contest beliefs of unfairness and respond with intergroup empathy bias because acknowledging structural racism would mean acknowledging an unjust system and their own complicity in entrenched systems of racial discrimination (Jones, 2004; Steyn, 2012; Jost, 2019).

Taken together, White participants’ outgroup perspective taking and empathic concern were compromised for the scenario portraying opposition to structural racism, potentially because such scenarios typically involve groups, may trigger intergroup threat, and challenge the fairness of the system. We believe prior perceptions about the continued impact of structural racism on Black people’s lives would influence the extent to which advantaged people respond with empathic concern in such situations. Study 3 tests this hypothesis.

## Study 3

Study 3 replicated the procedures of Study 2, but with the inclusion of questions to assess perceptions about the impact of interpersonal and structural racism, as in Study 1. We predicted that White participants’ perceptions of racism would again be skewed, with greater acknowledgment of the impact of interpersonal than structural racism in the lives of Black African people. Furthermore, we theorized that such predetermined notions about racism would meaningfully influence participants’ empathy responses (Bar-Tal and Halperin, 2011; Zaki, 2014), such that reduced perceptions of anti-Black structural racism would predict greater intergroup empathy bias in the scenario representing opposition to structural racism. Finally, we hypothesized that intergroup empathy bias would mediate the relationship between perceptions of anti-Black structural racism and willingness to attend an intergroup reconciliatory event.

### Methods

#### Participants

To determine the number of participants required to examine whether perceptions of anti-Black structural racism predict intergroup empathy bias, we conducted an *a priori* power analysis using G*Power 3.1 (Faul et al., 2007). This analysis indicated that we would need 68 participants for a multiple linear regression model with two predictors to detect a moderate effect (*f*^2^ = 0.15) with 80% power. We recruited 149 undergraduate students from lecture halls at the same historically White university as Study 2 to complete study procedures. Because the Black African participant sample was too small for analysis (*n* = 11), they were excluded, along with individuals self-identifying as Coloured, Indian, or foreign nationals (*n* = 53). The final sample consisted of 85 White participants (*M*_*age*_ = 19.24, SD_*age*_ = 0.91, 89% female, *M*_*yrs*_ _*edu*_ = 13.14, SD_*yrs*_ _*edu*_ = 0.77).

#### Measures

##### Reflective scenarios

The scenarios employed were similar to those in Study 2, with the following two exceptions. First, we changed the context of scenario 1 (structural racism) to that of a different university to determine whether White participants’ responses replicated when personal material costs were not at stake (i.e., their own institution). Second, we omitted scenario 3 (interpersonal racism) for the following reasons: (i) To reduce the amount of time to complete the questions and thus increase attention/interest of respondents. (ii) Because Black African participants reported high negative emotion (anger and frustration), in addition to empathic concern, in response to the Black target individual in this scenario (see Study 2, Supplementary Table 2). Since the scenarios served to examine whether White participants took the perspectives of outgroup individuals and responded with perspective taking and empathic concern in situations where Black African participants typically do, scenario 3 did not prove reliable in this regard. (iii) Scenario 3 was also more leading than scenario 2 in terms of an apparent “wrongdoer” and “victim,” thus requiring less perspective taking from participants. (iv) Finally, the perspective taking intercoder reliability of scenario 3 was the lowest. Scenario 2 (interpersonal injustice) remained unchanged and represented an interpersonal comparative context to scenario 1.

*Spontaneous perspective taking* for each target individual was assessed, as in Study 2, through two free-text questions following each scenario, i.e., “What do you think about [White/Black target behavior]?” The analyses proceeded in the same way, with perspective taking coded as present (1) or absent (0; Intercoder reliability: Kappa_*Scenario*__1_ = 0.77, Kappa_*Scenario*__2_ = 0.85, *p*s < 0.001).

##### Self-reported emotion

Emotions (frustration, compassion, anger, pride, shame, and anxiety) in response to the targets in each scenario were measured as in Study 2, from 1 (*not at all*) to 9 (*very much*). Empathic concern in Study 3 was operationalized in the same way as in Study 2 (i.e., compassion), and *negative emotion* consisted of a composite of frustration and anger ratings (αs > 0.85).

##### Perceptions about racism

Perceptions about the impact of interpersonal and structural forms of racism against Black African people (anti-Black racism during apartheid and today) and White people (anti-White racism today) were assessed as in Study 1. Responses were collected on Likert scales ranging from 1 (*not at all*) to 9 (*very much*). The difference between participants’ perceptions of anti-Black and anti-White racism today was again used as a proxy for perceived victimhood.

##### Intergroup interaction around historical oppression

A measure assessing participants’ willingness to attend an intergroup reconciliatory event was included. Participants were asked to respond to the question “If you have the opportunity to attend a cross-racial event where people share their experiences of discrimination during and after apartheid, would you be interested in going?” on a scale ranging from 1 (*not interested at all*) to *9* (*extremely interested*).

### Results

#### Perspective taking and empathic concern

As in Study 2, White participants’ perspective taking in response to Black target individuals were much less common for scenario 1 (structural racism: 38%) than scenario 2 (interpersonal injustice: 72%). Perspective taking for White target individuals in scenario 1 were again high (70%; Table 3).

**TABLE 3:** White participants’ (*N* = 85) perspective taking frequency scores for each scenario: Study 3.

	White target	Black target
**Scenario 1: Structural racism** PT = 0 PT = 1 Missing data	25 (30%) 58 (70%) 2	52 (62%) 32 (38%) 1
**Scenario 2: Interpersonal injustice** PT = 0 PT = 1 Missing data	51 (60%) 34 (40%) –	24 (28%) 61 (72%) –

Data presented are frequencies with percentage values in brackets. Missing data represent unanswered questions. PT = perspective taking, 0 = absent, 1 = present.

**FIGURE 3:**
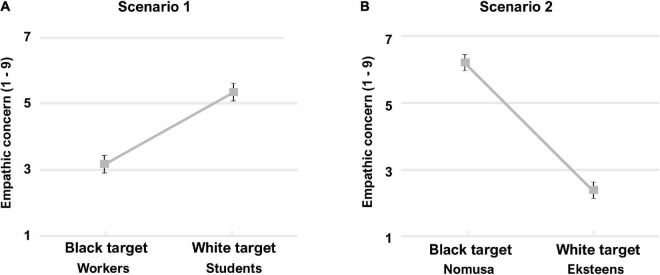
Study 3 White participants’ self-reported empathic concern ratings for **(A)** scenario 1 (structural racism) and **(B)** scenario 2 (interpersonal injustice). Error bars indicate standard error of the mean.

Regarding empathic concern, a paired-samples *t*-test indicated that White participants again displayed significant intergroup empathy bias for scenario 1: empathic concern was higher in response to White (*M* = 5.13, SD = 2.33) than Black (*M* = 3.15, SD = 2.30) target individuals, *t*(78) = –4.90, *p* < 0.001, *d* = 0.55 (Figure 3A). For scenario 2, White participants expressed more empathic concern toward the Black (*M* = 6.25, SD = 2.10) than White (*M* = 2.39, SD = 1.71) target individual, *t*(79) = 12.71, *p* < 0.001, *d* = 1.43 (Figure 3B). Other self-reported emotion ratings and statistical analyses are reported in the Supplementary material and Supplementary Table 4.

Four linear regressions confirmed that perspective taking scores again significantly predicted empathic concern for each target in each scenario (Scenario 1 Black target: β = 0.62, *p* < 0.001; Scenario 1 White target: β = 0.30, *p* = 0.008; Scenario 2 Black target: β = 0.24, *p* = 0.026; Scenario 2 White target: β = 0.56, *p* < 0.001).

#### Perceptions about racism

To compare perceptions of interpersonal and structural racism with those of Study 1, recalculated scores ranging from 1 to 100 are reported in Supplementary Table 5.

As in Study 1, participants perceived anti-Black structural racism (*M* = 3.42, SD = 2.17) to have significantly less of an impact than anti-Black interpersonal racism (*M* = 5.60, SD = 1.91) on Black African people today, *t*(84) = 9.94, *p* < 0.001, *d* = 1.08. Anti-Black structural racism (*M* = 8.75, SD = 0.80) was perceived to be of greater significance than anti-Black interpersonal racism (*M* = 8.26, SD = 1.30) during apartheid, *t*(83) = –4.06, *p* < 0.001, *d* = 0.44.

Regarding anti-White racism, participants perceived anti-White structural racism (*M* = 4.26, SD = 2.50) to have less impact than anti-White interpersonal racism (*M* = 4.82, SD = 2.41) on White people today, *t*(83) = 2.51, *p* = 0.014, *d* = 0.27. Importantly, we again observed heightened perceptions of racism experienced by the historically advantaged ingroup (anti-White racism) compared to the historically disadvantaged outgroup (anti-Black racism) for structural racism today, *t*(83) = 2.18, *p* = 0.031, *d* = 0.24. For interpersonal racism, however, Black African people were perceived to be more affected than White people today, *t*(84) = –2.22, *p* = 0.030, *d* = –0.24. Feelings of competitive victimhood for perceived structural racism thus also featured in this student sample.

To examine whether perceived structural racism against Black African people today would predict intergroup empathy bias in scenario 1 (opposition to structural racism), we conducted two simultaneous regressions. The first tested how well perceptions of anti-Black structural versus interpersonal racism today predicted intergroup empathy bias (ingroup empathic concern - outgroup empathic concern) for scenario 1, whereas the second tested how well these perceptions of racism predicted intergroup empathy bias for scenario 2 (see Table 4). As hypothesized, reduced perceptions of anti-Black structural racism significantly predicted greater intergroup empathy bias for scenario 1 (structural racism; *B* = –0.50, *p* = 0.016, boot SE = 0.21, 95% CI [–0.89, –0.11]). By comparison, reduced perceptions of anti-Black interpersonal racism significantly predicted intergroup empathy bias for scenario 2 (interpersonal injustice; *B* = –0.38, *p* = 0.042, boot SE = 0.21, 95% CI [–0.78, –0.01]).

**TABLE 4:** Simultaneous regressions predicting intergroup empathy bias as a function of perceptions of anti-Black structural and anti-Black interpersonal racism: Study 3.

Perceptions about racism	Scenario 1: Structural racism		Scenario 2: Interpersonal injustice	
		
	Intergroup empathy bias (*R*^2^ = 0.19, *p* < 0.001)		Intergroup empathy bias (*R*^2^ = 0.10, *p* = 0.018)	
		
	β	*B*	β	*B*
Anti-Black structural racism	–0.30	–0.50* [–0.89, –0.11]	–0.08	–0.10 [–0.24,0.43]
Anti-Black interpersonal racism	–0.20	–0.38 [–0.82, 0.04]	–0.27	–0.38* [–0.78, –0.01]

Data presented are beta values with 95% bootstrap confidence intervals (CIs) in brackets. Significant values are indicated in bold. *p < 0.05.

#### Intergroup interaction around historical oppression

**FIGURE 4:**
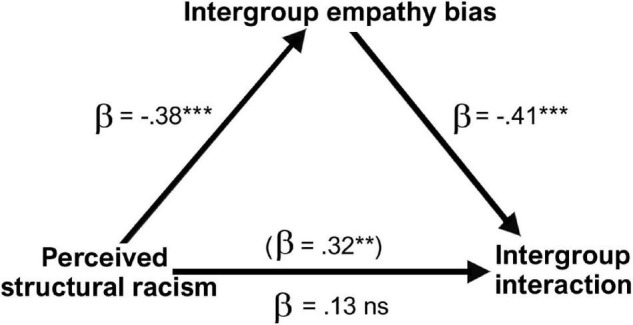
Intergroup empathy bias for situations of structural racism (scenario 1) fully mediated the effect of perceptions of anti-Black structural racism today on willingness to engage in intergroup interaction about historical oppression (*R*^2^ = 0.23, *p* < 0.001). Data presented are standardized β coefficients. **p < 0.01, ***p < 0.001.

To examine whether intergroup empathy bias, previously shown to directly motivate intergroup behavior (Bruneau et al., 2017), would mediate the effect of perceived anti-Black structural racism on willingness to engage in intergroup interaction around historical oppression, we conducted an exploratory mediation analysis. We conducted this analysis using Process v.4.1 Model 4 (Hayes, 2018). The model was specified with perceived anti-Black structural racism today as the independent variable, intergroup empathy bias for scenario 1 (structural racism) as the mediator variable, and intergroup interaction (*M* = 5.32, SD = 2.48) as the dependent variable (Figure 4). Indeed, the positive association between perceived anti-Black structural racism today and intergroup interaction (*B* = 0.36, *p* = 0.003, boot *SE* = 0.12, 95% CI [0.34,0.58]) was rendered non-significant when intergroup empathy bias was added as a mediator (*B* = –0.28, *p* < 0.001, boot *SE* = 0.09, 95% CI [–0.45, –0.10]), indicating full mediation (Indirect effect: *B* = 0.18, boot *SE* = 0.08, 95% CI [0.06,0.35]). Therefore, amongst participants with reduced perceptions of anti-Black structural racism today there was greater intergroup empathy bias for a situation portraying opposition to structural racism, which in turn, was associated with reduced willingness to engage in intergroup dialog around people’s lived experiences of historical oppression.

### Discussion

Study 3 replicated the effects observed in Studies 1 and 2, showing that the current sample perceived the impact of anti-Black structural racism to be less severe than that of anti-Black interpersonal racism, and that outgroup perspective taking and empathic concern in response to the scenario portraying structural racism was compromised. In addition, our analyses suggest that perceptions of anti-Black structural racism function to predict intergroup empathy bias in response to situations where Black people challenge an unjust system. By contrast, perceptions of anti-Black interpersonal racism predicted intergroup empathy bias in interpersonal contexts.

Because the setting of scenario 1 (opposition to structural racism) changed from Study 2 to 3, the results here suggest that White participant responses were not driven primarily by personal material interests. Rather, it provides support for our interpretation that opposition to structural racism could be symbolically threatening to White identity, because such resistance underscores conflicting beliefs between groups about the prevalence of structural racism (Riek et al., 2006). This interpretation is consistent with previous research suggesting that members of advantaged groups may experience threat when faced with the possibility of change to systems that benefit them, particularly when power relations are unstable (Saguy and Kteily, 2014). Such threat, in turn, often has the effect of raising support for ideologies that legitimize the racial *status quo* (Knowles et al., 2009; Jost et al., 2013). The present research extends these findings by showing that avoidance, denial, or lack of understanding of such realities of continuing structural racism is associated with reduced outgroup perspective taking and empathic concern when the unequal *status quo* is being challenged.

As in Study 1, perceptions of anti-Black interpersonal racism far exceeded those of anti-Black structural racism. Perceived anti-White interpersonal and structural racism were less elevated than in Study 1, however. This latter finding might reflect the younger age of the present student sample, as age has also previously been associated with more progressive outcomes in the South African context (Fourie and Verwoerd, 2022). Nevertheless, participants reported significantly greater perceptions of anti-White than anti-Black structural racism today, which suggests that competitive victimhood was also present in this younger sample.

Finally, Study 3 demonstrated that increased intergroup empathy bias in response to structural racism was associated with reduced willingness to engage in potentially challenging, yet important, cross-racial conversations about Black South Africans’ lived experiences of oppression during and after apartheid. Mediation analysis showed that participants with reduced perceptions of anti-Black structural racism responded with greater intergroup empathy bias, which in turn, was associated with reduced willingness to attend a cross-racial event to discuss historical injustices. Such encounters may allow for mutual understanding of the lived realities of people representing different historical perspectives, which has the potential to strengthen empathic connection and restore intergroup relations (Gobodo-Madikizela, 2008; Fourie et al., 2017). Consistent with previous findings, intergroup empathy bias thus served as a direct psychological motivator (barrier) of intergroup behavior (Bruneau et al., 2017).

## General discussion

Across three studies our data demonstrated significant discrepancies in White South African participants’ perceptions of anti-Black structural versus interpersonal racism, and that their reduced perceptions of anti-Black structural racism predicted difficulties in recognizing and empathizing with Black African people opposing structural racism. Intergroup empathy bias in response to the scenario depicting opposition to structural racism was furthermore associated with reduced willingness to engage in cross-racial discussions about historical injustices. While various motivated psychological processes and structural factors may contribute to limited recognition and/or insight into structural racism (Kraus et al., 2019), advantaged group members might also experience opposition to existing disparities as threatening to their moral integrity and privilege (Siem et al., 2013; Saguy and Kteily, 2014; Young and Sullivan, 2016). People of historically advantaged groups, such as White South Africans, might therefore be invested in contesting those who challenge current status arrangements, thereby disrupting perspective taking and empathic concern for them.

These findings are relevant because impaired outgroup perspective taking has far-reaching consequences in contexts that require recognition of intergroup inequalities (Todd et al., 2012; Todd and Galinsky, 2014). Appreciating another’s inner state endows them with moral rights and gives meaning to their actions (Waytz et al., 2010; Gray et al., 2012). Without perspective taking, it is a small leap toward perceiving outgroup members who advocate for structural change as a backward, monolithic mass (Kelman, 1973). Our results furthermore support the indelible relationship between recognizing experiences of structural racism and feeling empathic concern toward those who suffer its consequences.

The present research in the South African context corroborates existing accounts showing that the structural dimensions of racism are often minimized or silenced altogether by advantaged group members, despite its pervasive and visible effects (Milazzo, 2016; Cornell and Kessi, 2017; Salter et al., 2017; Albertus, 2019; Rucker and Richeson, 2021). Of significance, is that White participants’ reduced perceptions of anti-Black structural racism in our data were coupled with increased perceptions of anti-White structural racism; this while Whiteness continues to determine and mark privilege and power (Ratele and Laubscher, 2010; Chatterjee, 2019). These findings suggest that competitive victimhood poses a challenge for positive race relations in South Africa.

The needs-based model of reconciliation (Nadler and Shnabel, 2015) postulates that intergroup conflict differentially impacts the identities of victims and perpetrators, such that victims experience a need for empowerment, whereas perpetrators are motivated to restore their moral image. Motivations to restore these identity dimensions are particularly urgent in contexts where group disparity is salient and perceived as resulting from unjust structural violence (long-standing social arrangements that privilege some groups at the expense of others; Fiske et al., 2002; Siem et al., 2013).

Claiming victim status is a powerful strategy to restore a perpetrator group’s moral integrity. Indeed, the threatened identity and reversed moral standing of Whiteness post-apartheid provide ample impetus for White people to engage in competitive victimhood (Steyn, 2001). Various right-wing movements have capitalized on the notion that White South Africans, as a minority, are systematically oppressed and excluded by the ruling government−employing discursive strategies of victimhood and marginalization to bolster support (Van Zyl-Hermann, 2018). Such strategies, however, serve to preserve power and privilege of White people, while denying responsibility for the historical harm suffered by Black people (collectively speaking) and its continuing legacies (Verwey and Quayle, 2012; Noor et al., 2017).

The present work was not intended to reduce the complexities inherent to the racialized realities in South Africa (and beyond) to one underlying fracture, but rather to highlight one mechanism whereby *a priori* perceptions and motivated processes may give rise to empathic failures. If historically advantaged group members struggle to recognize or apprehend the magnitude of intergenerational structural racism and its impact on Black people’s lives, they are also unlikely to respond with empathic concern and allyship in situations where Black people challenge ongoing structural racism. Recent pushback against the international Black Lives Matter movement with slogans such as “White Lives Matter” or “All Lives Matter,” which devalue the racial injustices of the present political moment, serve as a case in point (Smith, 2017).

Our findings further suggest that intervening at the level of perceptions about structural racism should constitute a key mission for practitioners and policy makers to curb empathic failures or antipathy, and to inspire collective action. This should include instilling an understanding of racism firstly as large-scale structural and cultural forces that maintain racialized patterns of power and privilege, before it becomes interpersonal (Daumeyer et al., 2017; Salter et al., 2017; Kraus et al., 2019). Such messaging may include the continuing asymmetry in Black and White people’s experiences of institutional spaces, their unequal access to wealth and resources (e.g., health, education, justice), and the realities of low social mobility (Cornell and Kessi, 2017; Day and Fiske, 2017; McCall et al., 2017).

Understandings of structural racism should furthermore be embedded within a nuanced context of intergenerational structural violence, and advantaged group members’ role in perpetuating currently unjust realities, to refute assumptions that continuing inequality is a result of legitimate individual-level characteristics (Hetey and Eberhardt, 2018). These insights will additionally contribute to curbing perceptions of White victimhood (Green et al., 2017). Ultimately, the goal of interventions should not be to cultivate empathy in isolation. Rather, it should be to foster recognition of the systemic practices that individually and collectively reproduce structural racism in order to increase empathy-positive norms and garner support for and engagement in social justice (Zaki and Cikara, 2015; Durrheim and Dixon, 2018; Rothberg, 2019).

## Limitations

While one strength of the scenarios paradigm we employed is the assessment of spontaneous perspective taking *in vivo* (i.e., participants responded without overt instructions to take any party’s perspective), we also recognize potential limitations to it. First, because our study design was cross-sectional and correlational in nature, the causal direction of the observed associations between perspective taking and empathic concern in Studies 2 and 3 could not be established. Indeed, it could be that empathic concern predicted perspective taking (rather than the other way around). Nevertheless, we argue that the link from understanding a target’s perspective to empathic concern (compassion) for the target is most consistent with previous research, particularly in the absence of salient visual cues that might trigger concern, as in the present text-driven scenarios (Stietz et al., 2019).

Second, the scenarios were not balanced on all variables, such as the number of people involved, and the target-relevant information participants had (Todd and Galinsky, 2014). Though we have argued that situations of structural racism by nature would be more likely to involve groups than situations of interpersonal racism, scenario 1 (Studies 2 and 3) also dedicated somewhat more time to the White targets’ actions than the other scenarios (equal to that of the Black targets in scenario 1). Given the role of perspective taking in empathy production (Batson et al., 1997), this could have influenced participants’ perspective taking toward those White targets. We do not believe this somewhat unequal distribution of target-relevant information across the scenarios would have had a significant bearing on the principal contribution of this work, however, namely that advantaged group members responded with reduced perspective taking and empathic concern toward disadvantaged outgroup members challenging structural racism. Indeed, Black African participants’ responses showed the opposite effect in scenario 1. Still, future work of this nature should incorporate scenarios where target-relevant information is balanced.

Finally, we also note that a larger sample of Black African participants would give greater credence to our interpretation of their responses in Study 2 and should be pursued in future research. We were reassured by the fact that the present Black African sample was sufficiently powered to draw conclusions from their quantitative data, however. Furthermore, inspection of their qualitative scripts consistently showed Black African participants’ deeply felt concern for and understanding of the outsourced workers’ actions in scenario 1 as a protest against systemic racism and oppressive policies.

## Conclusion

Despite growing racialized inequality, White South African participants demonstrated reduced perceptions of anti-Black structural versus interpersonal racism, which not only contributed to reduced empathic concern for those opposing structural racism, but also predicted less willingness to engage in reconciliatory cross-racial dialogs. Such inaccurate perceptions of racism protect the racial *status quo* while allowing scope for competitive victimhood processes to flourish. We contend that without penetrating lay beliefs about the extent of structural inequities, it will remain hard for some White people to see beyond our privilege to empathize with and address the unequal legacies of apartheid and beyond.

## Positionality statement

Mindful that social identities can influence one’s approach to research and interpretation of data (Roberts et al., 2020), the authors wish to provide the reader with potentially relevant information about their backgrounds. With respect to race and gender, both MF and SM-B identify as White and female. With respect to nationality, MF identifies as South African, and SM-B identifies as United States American. While conscious of the intersectional complexity and heterogeneity within socially constructed racial categories, we believe referring to Black African and White people is warranted, without intending to oversimplify, given the legacies of systematic racial discrimination under colonialism and apartheid in South Africa.

## Data availability statement

The raw data supporting the conclusions of this article will be made available by the authors, without undue reservation.

## Ethics statement

The studies involving human participants were reviewed and approved by Stellenbosch University’s Humanities Research Ethics Committee. The participants provided their written informed consent prior to participating in this study.

## Author contributions

MF and SM-B contributed to conceptualizing the research. MF organized the data, performed the statistical analysis, and wrote and prepared the first draft of the manuscript. SM-B contributed to data interpretation and revision of the manuscript. Both authors read and approved the submitted version of the manuscript.

## Footnotes

1 Black here is used collectively to refer to all persons of color.

2 We wish to acknowledge that Coloured and Indian people also suffered systematic racial discrimination under the apartheid state.

3 We do not uncritically use the potentially problematic term anti-White racism, as racism does not occur in a vacuum separated from its historical context. Rather, our purpose for engaging this construct is to assess perceptions of White victimhood.
