# Supplementary material for: We cannot empathize with what we do not recognize: Perceptions of structural versus interpersonal racism in South Africa
Source: Front Psychol. 2022 Sep 28;13:838675. doi: 10.3389/fpsyg.2022.838675 (PMC9555212; doi:10.3389/fpsyg.2022.838675)
Supplement: Supplementary file 1 [file Table_1.DOCX]

# *Supplementary Material*

**Supplementary Methods: Study 1**

***Sample Information***

Data was collected by Springvale Online, an online marketplace panel service based in South Africa and established in 2005. The panel is one of the largest, most robust, and demographically diverse panels in South Africa, with > 40,000 respondents. The South African panel is regularly updated and randomly actively recruited, according to ESOMAR’s guidelines on conducting research over the internet. The following regions are included in the panel: Gauteng, Western Cape, KwaZulu-Natal, Eastern Cape, Mpumalanga, Free State, Limpopo, North West.

**Supplementary Results: Study 2**

***Perspective Taking***

Table S1

*Perspective Taking Examples in Response to Scenario 1, Scenario 2, and Scenario 3*

|  | White participants | | | Black African participants | | |
| --- | --- | --- | --- | --- | --- | --- |
|  |  |  |  |  |  |  |
| Scenario 1 (structural racism): Black target (workers) | | | | | | |
| Present  (PT = 1) | I think they were angered because they are trying to get a certain message across and the students cleaning it is telling the workers that their problems and beliefs are unimportant. | | | They were right, the workers were throwing mud as a protest action to show their disdain and dissatisfaction. And their protest action was directed at university management not “white students” who saw it fit to defend a demeaning system. The workers were resolute about being heard, and the white students were standing in the way of that. | | |
| Absent  (PT = 0) | I think the outsourced workers were out of line and extremely disrespectful. I feel as though the workers’ demonstration against the statue was uncalled for and not a way forward. | | | That was wrong of them. They destroyed something that does not even have anything to do with the protest. I feel frustrated and angry because it is not their property and they should get the message. | | |
| Scenario 1 (structural racism): White target (students) | | | | | | |
| Present  (PT = 1) | I think that they felt that their heritage was being disrespected and they had the right to act on that. I feel I would have done the same. | | | They decided to clean the statue maybe because they consider it as their heritage treasure as Mr J.H. Marais was white. I know how it feels when your work is undone. | | |
| Absent  (PT = 0) | I think that by cleaning the statue the students are invalidating what the workers stand for. Invalidating someone’s struggle isn’t something to be proud of. | | | I think it was a symbol of them perpetuating the violence and injustice. Thus it was a symbol of them siding with the former oppressive system. Such situations serve as a reminder that the majority of Stellenbosch refuses to acknowledge the struggles and concerns of the minority. | | |
| Scenario 2 (interpersonal injustice): Black target (Nomusa) | | | | | | |
| Present  (PT = 1) | I think it was extremely difficult for her because asking for money isn’t easy but it shows that she wants her daughter to experience a magical night amongst the previous disaster. She is in a tricky situation but still wants her daughter to enjoy herself. | | | She did what any mother would do to ensure her child experiences the best matric farewell regardless of their current living circumstances. I feel compassion for her as a hard working mother who was in great need. | | |
| Absent  (PT = 0) | I think it is a little much as it is not a life or death situation whereas the house burning down is. It’s one thing to ask for financial support for the accident and another to ask to pay for someone’s dress. | | | It was very ungrateful of her. | | |
| Scenario 2 (interpersonal injustice): White target (Eksteens) | | | | | | |
| Present  (PT = 1) | The fact that it was very expensive and technically non-essential made it a bit of an imposition. Because it was an expensive non-essential item, I understand denying the request. | | | It was their choice to make. Maybe they thought it unimportant, as it was a luxury not a need. They had every right to say no. | | |
| Absent  (PT = 0) | I do not approve of this as this sum of money was most probably of little significance to them. They have the means but were not willing. | | | It is saddening, considering the assistance she provides to them. This expresses the inequality that is still prominent in society along racial lines. If one has the means to assist another who is less advantaged, why should one not do so? | | |
| Scenario 3 (interpersonal racism): Black target (Zoliswa) | | | | | | |
| Present  (PT = 1) | I think it’s well justified. If you can’t enjoy your sport, don’t do it just for the prestige. She made a decision in her own best interest and I honour her for that, even if others don’t understand. | | | It’s ok because feeling unwanted on a team can hinder her performance. I have felt it myself before. I can relate. | | |
| Absent  (PT = 0) | She overreacted, if she was good enough she would feel she deserves to be there. She gave up on her dreams too easily | | | Irrational. Her abilities should not be determined by her skin color. It is very sad to see how she had to stop her dreams because of her skin color. She’s giving the whites too much power over her. | | |
| Scenario 3 (interpersonal racism): White target (players) | | | | | | |
| Present  (PT = 1) | Justified, many white players lose spots they deserve due to quota. I understand as nowadays, many white players lose out on what their skills deserve due to quota, but there is no justification for the racial hostility. | | | ⸺ | | |
| Absent  (PT = 0) | Utterly shameful. It is disgusting that their attitude led to such a consequence. | | | It could mean different things based on social behavior, one would regard this as racism or discrimination. Such behavior is shocking in the current South Africa. | | |
|  |  |  |  |  |  |  |

*Note*. PT = perspective taking, 0 = absent, 1 = present.

***Scenario 1: Example Qualitative Responses***

Black African participants: Black target (workers)

- - I think it’s fair because they are trying to be heard through their actions but the students are erasing their efforts, basically belittling their cries. The workers did not back down and that made me feel proud.
  - I think they felt that their voices were not being heard and marked as irrelevant.
  - I think they are justified in the anger and continuous throwing of mud because it would seem as if the students are trying to silence the workers. The workers must have felt disrespected.
  - It was an act of desperation, feeling that their issue was being ignored.
  - They were angry and felt that this was the only way to be heard.
  - I think it shows their determination about their cause, continuing to act until actual talk is initiated. Compassion because I understand where they are coming from.
  - I think their response was correct, as they were having a battle with the University not the students
  - They perhaps felt agitated; as maybe they felt as if their act of frustration was being ignored.
  - Why they outsourced where they should be insource by the university, they should be permanent.
  - It was an act of desperation, feeling that their issue was being ignored. I feared for the consequences of their actions.
  - Owing to the experiences the outsourced workers may have, it is quite saddening that the only means by which they could overtly express their feelings was the above. It shows how deeply entrenched the effects of racism are, even in current society.
  - They were right, the workers were throwing mud as a protest action to show their disdain and dissatisfaction. And their protest action was directed at University management not “White students” who saw it fit to defend a demeaning system. Because the workers were resolute about being heard, and the white students were standing in the way of that.
  - They are minimalizing the workers protest and making it seem futile. Both compassion and shame. I pity that they chose to disregard the colonialist in post-colonial/apartheid.
  - It definitely cannot be condemned. The workers have every right to be angered by being marginalised by the colonial figure. They did not want to make their “movement” seem futile. Three emotions are interconnected. They must have felt so angered by the condoning of the J.S Marais legacy. It is difficult to live in post-apartheid /colonial SA if the people living amongst you continue to praise colonists. It is sad to witness hierarchies.
  - Trying to maintain their point. Protesting is an expression of their anger in a way that grabs attention. Having that undermined does not change the fact that anger exists nor the need to express it that way. Protesting is a last resort. This action means that change has not been established using other means.

Black African participants: White target (students)

- - They show that the statue is someone that belongs to their group ethnic which is white privilege.
  - They act like spoiled brats and superheroes who are racist.
  - The students probably thought they were doing the right thing, but the workers had their reasons and the students should have let the workers make their statements. The anger and anxiety are because the students would only be increasing the action and the issues of the workers may never be dealt with. They are meddling.
  - I think that’s what they think is right, but I feel they are being ignorant to the reason why the workers are throwing mud – it’s not just to destroy property for no reason.
  - I think they should have left the University to deal with the issue, it did not concern them in any way. Their behaviour was unacceptable.
  - They embrace the statue because that person is in their ethnic group. I felt bad for the workers because they were expressing themselves and their opinion was not considered.
  - I don’t know their intentions but it may have swept the real issue at hand under the rug as the damage caused had been ‘fixed.’ I relate with the worker’s reason for doing it.
  - It’s obviously their thing i.e. they felt the need to clean the statue because it meant something to them. It’s disturbing, the whole situation is disturbing.
  - They simply do not understand the plight of the workers and have no historical consciousness of the politics of process. Because what they did was simply disrespectful and showed no regard for the plight of the poor.
  - Undermining the workers complaints and protest. Protecting the legacy of a dead racist over the rights of living, hardworking people is disgusting.
  - They were very arrogant because it was not their place to clean up the statue. Their actions angered the workers because the fight was between the workers and RMT, NOT the white students. I felt very annoyed by their arrogance because whatever was happening had nothing to do with them.

White participants: Black target (workers)

- - Very immature. Anger, very childish action and response. What will throwing mud help?
  - They were disrespectful and could’ve approached the problem in a more constructive way. They acted out, yet their issues shouldn’t be with the students but with the university itself.
  - I think it was immature and that disruptive protest is unnecessary.
  - They were simply acting out of anger but their actions were disgraceful towards heritage. It made me angry and shameful because they were disrespectful.
  - I believe it was immature but understandable and perhaps predictable.
  - I feel that the damaging or destruction of any kind is wrong, it is frustrating that people think it is okay.
  - Very immature, should have spoken it out. Anger, very childish action and response.
  - Childish response that would not achieve anything.
  - I get that he was a bad man but I don’t think destroying and vandelizing property is the right thing to do.
  - The fact that the workers resorted to vandalism to voice their unhappiness is wrong in the first place. The fact that they were angered about the fact that someone cleaned it up is silly.
  - I think they should have ceased their mud throwing after they saw the commitment of the students. The workers should have stayed professional instead of throwing mud.

White participants: White target (students)

- - I believe it was the honourable response, as they were displaying pride in the statue. I was proud in that they did not respond in anger or revenge
  - I think it was courageous and inspiring if their intentions weren’t to propagate further anger or protest.
  - They tried to do the right thing and avoid violence, but they could’ve gone further and reported the problem or approach the workers.
  - I think that they felt that their heritage was being disrespected and they had the right to act on that. I feel I would have done the same.
  - It is their own choice what they want to do, and if that is to clean the statue. Then so be it. Their choice should be respected. They felt that it was their place to respect our heritage no matter what form it is in. Being a proud South African heritage is important to me.
  - Mature decision. Pride, it was good citizenship.
  - I agree with what they did, they cleaned out a statue to keep the university grounds clean and restore a university heritage. I was proud that they stood up for their views.
  - I think it was good, as they were cleaning up and helping with damaged property.
  - I think it was correct of them, they were taking care of a public nuisance.
  - The decision to clean the statue was simply a peaceful way to go against the protests. They were just cleaning up a mess made by other people, not a violent retaliation.
  - I think it was the right thing to do as throwing mud on the statue is disrespectful. I feel like it would be such a chore and hard to find the time to clean the statue.

***Self-reported Emotion***

Table S2

*Self-reported Emotion Ratings from 1 (Not at All) to 9 (Extremely): Study 2*

|  | White participants | | | |  | Black African participants | | | |
| --- | --- | --- | --- | --- | --- | --- | --- | --- | --- |
|  | White target | | Black target | |  | White target | | Black target | |
|  | *Mean* | *SD* | *Mean* | *SD* |  | *Mean* | *SD* | *Mean* | *SD* |
| *Scenario 1: Structural racism* | | | | |  |  |  |  |  |
| Empathic concern | **5.18** | 2.23 | 2.95 | 2.02 |  | 3.30 | 2.49 | **5.48** | 2.73 |
| Negative emotion | 2.33 | 1.82 | **5.52** | 2.21 |  | 4.06 | 2.81 | 2.74 | 2.47 |
| Pride | **5.29** | 2.74 | 1.64 | 1.34 |  | 3.00 | 2.70 | 3.88 | 2.92 |
| Shame | 2.30 | 2.04 | 4.11 | 2.60 |  | 2.39 | 2.02 | 3.30 | 2.98 |
| Anxiety | 2.82 | 2.10 | 3.58 | 2.38 |  | 3.39 | 2.44 | 2.91 | 2.68 |
| *Scenario 2: Interpersonal injustice* | | | | |  |  |  |  |  |
| Empathic concern | 2.40 | 1.96 | **5.85** | 2.32 |  | 1.83 | 1.43 | **6.28** | 2.37 |
| Negative emotion | **3.99** | 2.37 | 1.81 | 1.24 |  | **4.33** | 3.16 | 1.91 | 1.59 |
| Pride | 1.40 | 1.03 | 2.52 | 1.99 |  | 1.78 | 2.15 | 2.71 | 2.26 |
| Shame | 3.66 | 2.56 | 2.20 | 2.03 |  | 3.96 | 2.90 | 3.42 | 2.67 |
| Anxiety | 2.11 | 1.68 | 2.10 | 1.80 |  | 2.04 | 2.06 | 2.56 | 2.22 |
| *Scenario 3: Interpersonal racism* | | | | |  |  |  |  |  |
| Empathic concern | 1.59 | 1.36 | **5.19** | 2.56 |  | 2.13 | 2.60 | **5.35** | 2.96 |
| Negative emotion | **7.01** | 1.83 | **4.80** | 2.34 |  | **8.04** | 1.23 | **5.89** | 2.41 |
| Pride | 1.26 | .95 | 2.07 | 1.95 |  | 1.05 | .21 | 2.65 | 2.76 |
| Shame | 5.92 | 2.56 | 3.31 | 2.45 |  | 5.83 | 3.31 | 3.74 | 2.82 |
| Anxiety | 2.75 | 2.23 | 2.60 | 2.11 |  | 3.68 | 3.39 | 3.43 | 2.92 |

*Note*. Emotions most highly rated in response to each target are indicated in boldface. Negative emotion is a composite measure consisting of ratings of frustration and anger.

Self-reported emotion ratings (Table S2) were subjected to repeated-measures ANOVAs. In instances where the assumption of sphericity was violated, the degrees of freedom were adjusted using Greenhouse-Geisser epsilon corrections.

For scenario 1 (structural racism), repeated-measures ANOVAs confirmed that significant differences existed between White participants’ self-reported emotion ratings in response to the White and Black target individuals, respectively, *F*s > 60.80, *p*s < .001, ƞ^2^ > .33. Planned contrasts showed that White participants’ responses of pride and empathic concern were significantly higher than all other emotion ratings in response to the White target individuals (students) (*p*s < .001, *r*s > .61), while ratings of pride and empathic concern did not differ (*p* = .62, *r* = .04). White participants expressed predominantly high negative emotion in response to the Black target individuals (workers), however (*p*s < .001, *r*s > .48).

For Black African participants, there were no significant differences in reported emotion in response to the White target individuals, *F*(2.44, 53.57) = .38, *p* = .54, ƞ^2^ = .02, *ε =* .61. A repeated-measures ANOVA indicated that there were significant differences in reported emotion in response to the Black target individuals, *F*(2.30, 48.31) = 2.76, *p* = .03, ƞ^2^ = .12, *ε =* .58. Planned contrasts indicated that empathic concern was the emotion most highly felt in response to the Black target individuals (*p*s < .05, *r*s > .41).

For scenario 2 (interpersonal injustice), repeated-measures ANOVAs confirmed that White participants’ self-reported emotion ratings differed significantly in response to Black and White target individuals, respectively, *F*s > 41.50, *p*s < .001, ƞ^2^ > .26. Planned contrasts showed that empathic concern was most highly felt in response to the Black target individual (Nomusa) (*p*s < .001, *r*s > .79). By contrast, negative emotion was most commonly expressed in response to the White target individuals (Eksteens) (*p*s < .01, *r*s > .28).

Self-reported emotion ratings of Black African participants followed a similar pattern for scenario 2. Repeated-measures ANOVAs confirmed that Black African participants’ self-reported emotion ratings differed significantly in response to the Black and White target individuals, respectively, *F*s > 6.61, *p*s < .001, ƞ^2^ > .23. Planned contrasts showed that empathic concern was the emotion most highly felt in response to the Black target individual (Nomusa) (*p*s < .001, *r*s > .75). By contrast, negative emotion was most commonly expressed in response to the White target individuals (Eksteens) (*p*s < .01, *r*s > .50).

For scenario 3 (interpersonal racism), repeated-measures ANOVAs confirmed that White participants’ self-reported emotion ratings differed significantly in response to the Black and White target individuals, respectively, *F*s > 52.60, *p*s < .001, ƞ^2^ > .32. Planned contrasts showed that empathic concern and negative emotion were rated significantly higher than all other emotions in response to the Black target individual (Zoliswa) (*p*s < .001, *r*s > .53), while empathic concern and negative emotion did not differ (*p* = .22, *r* = .11). By contrast, negative emotion alone was most highly expressed in response to the White target individuals (players) (*p*s < .001, *r*s > .46).

Self-reported emotion ratings of Black African participants followed a similar pattern for scenario 3. Repeated-measures ANOVAs confirmed that Black participants’ self-reported emotion ratings differed significantly in response to the Black and White target individuals, respectively, *F*s > 6.00, *p*s < .001, ƞ^2^ > .21. Planned contrasts again showed that both empathic concern and negative emotion was expressed most prominently in response to the Black target individual (Zoliswa) (*p*s < .01, *r*s > .55), while empathic concern and negative emotion did not differ (*p* = .54, *r* = .13). By contrast, negative emotion was most highly expressed in response to the White target individuals (players) (*p*s < .01, *r*s > .58).

***Perspective Taking and Empathic Concern***

Table S3

*Logistic Regressions Predicting Perspective Taking (Absent/Present) as a Function of Empathic Concern and Participant Race: Study 2*

|  | 95% CI for exp *b* | | | |
| --- | --- | --- | --- | --- |
|  | *B* (*SE*) | Lower | Exp *b* | Upper |
| Scenario 1: Black target (workers) | *R*^2^ = .47, Model χ^2^(2) = 59.33, *p* < .001 | | | |
| Empathic concern | .57*** (.113) | 1.41 | 1.76 | 2.19 |
| Participant race | -1.99** (.68) | .04 | .14 | .52 |
| Constant | 1.13 (1.35) |  | 3.09 |  |
| Scenario 1: White target (students) | *R*^2^ = .24, Model χ^2^(2) = 18.34, *p* < .001 | | | |
| Empathic concern | .32* (.14) | 1.05 | 1.38 | 1.82 |
| Participant race | 1.50* (.63) | 1.31 | 4.49 | 15.39 |
| Constant | -2.07* (1.04) |  | .13 |  |
| Scenario 2: Black target (Nomusa) | *R*^2^ = .30, Model χ^2^(2) = 27.55, *p* < .001 | | | |
| Empathic concern | .55*** (.12) | 1.37 | 1.73 | 2.17 |
| Participant race | -.56 (.86) | .11 | .57 | 3.09 |
| Constant | -.01 (1.73) |  | .99 |  |
| Scenario 2: White target (Eksteens) | *R*^2^ = .12, Model χ^2^(2) = 12.93, *p* = .002 | | | |
| Empathic concern | .37** (.12) | 1.14 | 1.45 | 1.84 |
| Participant race | .35 (.47) | .56 | 1.42 | 3.58 |
| Constant | -1.05 (.89) |  | .35 |  |
| Scenario 2: Black target (Zoliswa) | *R*^2^ = .07, Model χ^2^(2) = 7.65, *p* = .022 | | | |
| Empathic concern | .18** (.07) | 1.05 | 1.20 | 1.38 |
| Participant race | .21 (.47) | .49 | 1.23 | 3.12 |
| Constant | -1.10 (.97) |  | .33 |  |
| Scenario 2: White target (players) | *R*^2^ = .43, Model χ^2^(2) = 20.73, *p* < .001 | | | |
| Empathic concern | 1.03*** (.27) | 1.64 | 2.79 | 4.73 |
| Participant race | 22.17 (6207.94) | .00 | .00 | .00 |
| Constant | -49.42 (1241.87) |  | .00 |  |

*Note*. Participant race was coded as 1 = Black African, 2 = White. *R*^2^ = Nagelkerke

**p <* .05. ***p* < .01. ****p* < .001.

**Supplementary Results: Study 3**

***Self-reported Emotion***

Table S4

*Self-reported Emotion Ratings from 1 (Not at All) to 9 (Extremely) (White participants: N = 85): Study 3*

|  | Scenario 1: Structural racism | | | |  | Scenario 2: Interpersonal injustice | | | |  |
| --- | --- | --- | --- | --- | --- | --- | --- | --- | --- | --- |
|  | White target | | Black target | |  | White target | | Black target | | |
|  | *Mean* | *SD* | *Mean* | *SD* |  | *Mean* | *SD* | *Mean* | *SD* | |
| Empathic concern | **5.22** | 2.35 | 3.10 | 2.28 |  | 2.41 | 1.72 | **6.22** | 2.08 | |
| Negative emotion | 2.43 | 2.05 | **4.89** | 2.34 |  | **3.91** | 2.27 | 1.82 | 1.17 | |
| Pride | 4.60 | 2.80 | 1.72 | 1.21 |  | 1.18 | 0.60 | 2.29 | 2.08 | |
| Shame | 2.00 | 1.81 | 3.41 | 2.71 |  | 3.22 | 2.34 | 2.02 | 1.93 | |
| Anxiety | 2.55 | 1.89 | 3.26 | 2.44 |  | 2.21 | 1.93 | 2.01 | 1.79 | |

*Note*. Emotions most highly rated in response to each target are indicated in boldface. Negative emotion is a composite measure consisting of ratings of frustration and anger.

Self-reported emotion ratings (Table S4) were subjected to repeated-measures ANOVAs. In instances where the assumption of sphericity was violated, the degrees of freedom were adjusted using Greenhouse-Geisser epsilon corrections.

For scenario 1 (structural racism), repeated-measures ANOVAs confirmed that significant differences existed between White participants’ self-reported emotion ratings in response to the White and Black target individuals, respectively, *F*s > 21.80, *p*s < .001, ƞ^2^ > .23. Planned contrasts showed that White participants’ responses of empathic concern were significantly higher than shame, anxiety, and negative emotion ratings in response to the White target individuals (students) (*p*s < .001, *r*s > .62), and also higher than pride (*p* = .02, *r* = .27). White participants’ negative emotion ratings were higher than any other emotion in response to the Black target individuals (workers), however (*p*s < .001, *r*s > .44).

For scenario 2 (interpersonal injustice), repeated-measures ANOVAs confirmed that White participants’ self-reported emotion ratings differed significantly in response to the Black and White target individuals, respectively, *F*s > 28.00, *p*s < .001, ƞ^2^ > .27. Planned contrasts showed that empathic concern was the emotion most highly felt in response to the Black target individual (Nomusa) (*p*s < .001, *r*s > .87). By contrast, negative emotion was most commonly expressed in response to the White target individuals (Eksteens) (*p*s ≤ .01, *r*s > .36).

***Perceptions about Racism***

Table S5

*Participants’ Perceptions About Interpersonal and Structural Racism: Study 3*

|  | Interpersonal racism | | Structural racism | | Comparison of Means (*p*) |
| --- | --- | --- | --- | --- | --- |
|  | *M* | *SD* | *M* | *SD* |  |
| During apartheid |  |  |  |  |  |
| Anti-Black | 91.78 | 14.44 | 97.22 | 9.12 | < .001 |
| Today |  |  |  |  |  |
| Anti-Black | 62.22 | 21.11 | 38.04 | 24.11 | < .001 |
| Anti-White | 53.56 | 26.78 | 47.33 | 27.78 | .014 |
|  |  |  |  |  |  |

*Note.* Ratings ranged from 1 (*Not at All*) to 9 (*Extremely*) and were recalculated to range from 1 to 100.
